# Gut microbiome diversity and composition is associated with Hypertension in women

**DOI:** 10.1097/HJH.0000000000002878

**Published:** 2021-09-01

**Authors:** Panayiotis Louca, Ana Nogal, Philippa M. Wells, Francesco Asnicar, Jonathan Wolf, Claire J. Steves, Tim D. Spector, Nicola Segata, Sarah E. Berry, Ana M. Valdes, Cristina Menni

**Affiliations:** a Department of Twin Research and Genetic Epidemiology, King’s College London, London, England, SE1 7EH, United Kingdom; b Department CIBIO, University of Trento, Trento, Italy; c Zoe Global Limited, London SE1 7RW, UK; d Department of Nutritional Sciences, King’s College London, London SE1 9NH, United Kingdom; e Academic Rheumatology Clinical Sciences Building, Nottingham City Hospital, University of Nottingham, United Kingdom

**Keywords:** Hypertension, gut microbiome, diversity

## Abstract

**Objectives:**

Animal studies support a role for the gut microbiota in hypertension development, but large human studies are lacking. Here we investigated the relationship between hypertension prevalence and gut microbial composition in two cohorts.

**Methods:**

We included 871 unrelated TwinsUK females with faecal microbiome data (16s rRNA gene sequencing). Multivariable linear models adjusted for age, age^2^ and BMI as well as MiRKAT models, were used to estimate the association of hypertension with alpha- and beta-diversity metrics. To identify taxa associated with hypertension, a generalized additive model for location scale and shape was computed adjusting for covariates and multiple testing. Results were replicated in 448 women from PREDICT-1

**Results:**

We found that measures of alpha diversity are significantly lower in hypertensive cases (Beta[95%CI] = -0.05[-0.095, -0.004], p=0.03) and a significant association between beta diversity and hypertension (FDR < 0.05). We identified and replicated 2 genera associated with hypertension. The genus, *Ruminiclostridium 6* was less abundant in hypertension cases (meta-analysis[95%CI] = -0.31[-0.5; -0.13], p = 1x10^-3^). The uncultured microbe *Erysipelotrichacea-UCG003* was more abundant in hypertensive cases (meta-analysis[95%CI] = 0.46[0.3, 0.62], p=1x10^-4^). We genomically analysed the 16s rRNA sequence and established a 100% identity match with the 16s rRNA sequence of the genus *Faecalibacillus*. We functionally annotated *Ruminiclostridium*, identifying 83 metabolic pathways, including pathways previously linked to blood pressure regulation.

**Conclusion:**

In this large human observation, we show that gut microbiome diversity and composition are associated with hypertension. Our results suggest that targeting the microbiome may be a novel means to prevent or treat hypertension.

## Introduction

Hypertension is the most prevalent modifiable risk factor for cardiovascular morbidity and mortality affecting >1.3 billion people worldwide [1]. Given the burden of hypertension, any strategy that will improve blood pressure (BP) control will have major public health benefits [2]. The causation of hypertension is multifactorial [3], influenced by a host of genetic and environmental factors, including poor diet, obesity, inactivity and smoking, in addition to interactions between these factors [3]. The human microbiota comprises 10-100 trillion symbiotic microbial cells harboured by each person and is acquired from the environment starting at birth [4,5]. The gut microbiome, i.e. the community of microbes in the gastrointestinal tract, has been recently shown to be an important determinant of inflammation [6], obesity [7], type-2 diabetes [8] and arterial stiffness [9–11], all of which contribute to the risk of hypertension [12]. Moreover, animal studies suggest that gut microbes may act on downstream cellular targets, directly contributing to the pathogenesis of hypertension [9,13]. Recently human studies have identified lower gut microbiome diversity [13–16] and specific gut microbes associated with hypertension[17,18] In a recent review on gut microbiome composition and hypertension, Verhaar and collaborators[18] indicated the negative role of Gram-negative microbiota including *Klebsiella*, *Parabacteroides*, *Desulfovibrio*, and *Prevotella* and the possible neutral/protective role of *Ruminococcaceae*, *Roseburia*, and *Faecalibacterium*[18].Previous research into the gut microbial composition to hypertension also highlights the enrichment of *Eubacterium*[19], *Lactobacillus* [14], *Megasphaera*[14], and *Rothia*[14], and the depletion of *Bacteroides[19], Enterococcus[20], Oscillibacter[16]*. Moreover, a large study on 7,928 individuals showed the periodontal microbiome, including *C. rectus, V. parvula and P. melaninogenica*, to be involved in blood pressure regulation, suggesting the possible pro-hypertensive role of bacteria and bacterial products throughout the gastrointestinal tract and the mouth[21,22]. However, human studies are typically small and therefore underpowered, lack independent replication and there is a paucity of population-based evidence. Here we first investigated the association between hypertension, and gut microbiome composition. We looked at the relationship between hypertension and the loss of microbiome composition and with specific genera in 871 people from TwinsUK. We then replicated our results in an independent population and further genomically characterise the hypertension-associated microbes using their 16s rRNA sequences.

## Methods

### Design and study population

#### Discovery cohort

Study subjects were female unrelated individuals enrolled in the TwinsUK registry, a national register of adult twins recruited as volunteers without selecting for any particular disease or traits [23] Here we analysed data from 871 unrelated females from TwinsUK [23]. Twins provided informed written consent and the study was approved by St. Thomas’ Hospital Research Ethics Committee (REC Ref: EC04/015). Data relevant to our analysis includes BP, antihypertensive drug use, BMI, age and gut microbiome composition assessed using 16s rRNA, as described below

#### Replication cohort

The replication cohort consisted of an independent sample of 448 females from the UK based PREDICT 1 study with analogous data. The PREDICT-1 study [24] was a single-arm nutritional intervention conducted between June 2018 and May 2019. Study participants were healthy individuals (thus eliminating potential confounders brought about by the presence of infections or other comorbidities) aged between 18-65 years recruited from the TwinsUK registry [23], and the general population using online advertising. Participants attended a full day clinical visit consisting of test meal challenges followed by a 13-day home-based phase, as previously described [24].

### Measurements and variables

#### Blood pressure

Blood pressure was measured by a trained nurse using either the Marshall mb02, the Omron Mx3 or the Omron HEM713C Digital Blood Pressure Monitor performed with the patient in the sitting position for at least 3 minutes. At each visit, the cuff was placed on the subject’s arm so that it was approximately 2–3 cm above the elbow joint of the inner arm, with the air tube lying over the brachial artery. The subject’s arm was placed on the table or supported with the palm facing upwards, so that the tab of the cuff was placed at the same level of the heart. Triplicate measurements were taken with an interval of approximately 1 min between each reading, with mean of second and third measurements recorded.

Subjects were classified into two groups based on their BP level, age and use of BP lowering drugs: hypertension cases (<60 years of age, with systolic BP (SBP) ≥140 mmHg, or diastolic BP (DBP) ≥90 mmHg, or currently using anti-hypertensive drugs, or started using before 60 years); and controls (if age >50 years, SBP ≤120 mmHg and DBP ≤80 mmHg, and not on BP lowering medication, or if age ≤50, SBP ≤115 mmHg and DBP ≤80 mmHg and not on BP lowering medication).

#### Gut microbiome composition

Gut microbiome composition was determined by 16S rRNA gene sequencing as previously described [4]. Briefly, the V4 region of the 16S rRNA gene was amplified and sequenced on Illumina MiSeq. 16S sequences were demultiplexed in QIIME. Amplicon sequence variants (ASV) were then generated using the DADA2 package in R using the pipeline described elsewhere [25]. Observed Amplicon sequence variants (ASV) and beta diversities were computed using the R packages “vegan” [26] and “microbiome” [27].

### Statistical Analysis

Statistical analysis was done using R 4.0.2.

We used general linear models with a quasi-Poisson distribution to investigate associations between observed ASVs and hypertension. The association between beta-diversity metrics and hypertension was assessed via MiRKAT tests[28]. After grouping ASV at genus level, to identify taxa associated with hypertension, a generalized additive model for location, scale and shape (GAMLSS) fitted with the zero-inflated beta distribution (BEZI) was computed using the R package “gamlss” [29]. The GAMLSS-BEZI model is a two-component mixture model including a zero-model accounting for excess zeros and a count model to capture the remaining component by beta regression, allowing for overdispersion effects. The first component of this mixture model is linked by the nu parameter that models the probability at zero, the second component is indexed by the mu and sigma parameters, respectively the mean and precision parameters. Likelihood-ratio tests between the null model (covariates only) and full models (covariates and hypertension) were performed (FDR<0.01). We adjusted for age, age^2^, BMI and multiple testing using false discovery rate (FDR<0.05).

We replicated significant genera (at p <0.05) in PREDICT 1 and results were combined using an inverse variance random effect meta-analysis [30].

### Genomic characterisation of hypertension – associated microbes

All genomes isolated from the human gut belonging to the hypertension –associated microbes (*Ruminiclostridium* and the *Erysipelotrichaceae)* families were downloaded from the Unified Human Gastrointestinal Genome catalogue [31] (and RefSeq data set (January, 2021) for the *Erysipelotrichaceae* family). CheckM v1.1.3 [32] was then ran using the “lineage_wf” workflow to estimate genomic completeness and contamination. For *Erysipelotrichaceae* genera we used high quality genomes (<3% contamination, >95% completeness) of representative species. Whereas for the *Faecalibacillus* genus we used the genomes of all existent species.

#### Evolutionary relationship of the Erysipelotrichaceae family

As *Erysipelotrichaceae UCG003* was uncultured, we performed a genomic evolutionary analysis to investigate close relationships with other microbes. We predicted the 16S rRNA sequences of the representative *Erysipelotrichaceae* species using barrnap v0.9 (http://www.vicbioinformatics.com/software.barrnap.shtml). The predicted sequences together with the 16S rRNA sequence of *Erysipelotrichaceae UCG-003* were then aligned using MUSCLE v3.8.1551 [33]. From which, we estimated the maximum-likelihood phylogeny with PhyML v3 [34] implemented in SeaView v5.0.4 [35], using the general time reversible (GTR) model of evolution, with optimized number of variant sites, nucleotide equilibrium frequencies and across site rate variation, and 100 bootstrap replicates. The constructed phylogenetic tree was then visualized using Interactive Tree Of Life (iTOL) [36]. Additionally we queried the 16S rRNA sequence of *Erysipelotrichaceae UCG-003* in the RDP classifier [37] and BLASTn [38]. In BLASTn, the search was filtered by organism (*Erysipelotrichaceae* [taxid:128827]).

#### Prediction of the functional capabilities of Ruminiclostridium

To perform the functional analysis of *Ruminiclostridium*, we filtered genomes by a higher standard (>95% completeness, <1% contamination and <300 contigs). Missing sample accession numbers were obtained from the Sequence Read Archive (SRA) database of the NCBI, and the genomes from sample identifiers not found in the NCBI were discarded. Duplicated genomes were removed, keeping the genome with the highest N50 value. After filtering, we ran Prokka v1.12 [39] in 565 remaining *Ruminiclostridium* genomes while specifying the genus (parameter --genus) to retrieve the gff files. Specifically, enzyme commission numbers were extracted to identify the Encyclopedia of Genes and Genomes (KEGG) [40] pathways using MiniPath (Minimal set of Pathways) (version: September, 2020) [41]. All the KEGG identifiers related to environmental information processing and organismal systems were removed. For each metabolic pathway, the number of genomes that presented such pathway was calculated.

## Results

We included 871 unrelated females (397 cases, 474 controls) from TwinsUK, aged 56 (±11.3), and overweight, with an average BMI of 26 Kg/m^2^ (±5) and we replicated our results in an independent sample of 448 females (57 cases, 391 controls) from the PREDICT-1 study, aged 44.8 (±12.1) with an average BMI of 25.1 Kg/m^2^ (±5.1). Descriptive characteristics of the study populations are shown in Table 1.

**Figure 1:**
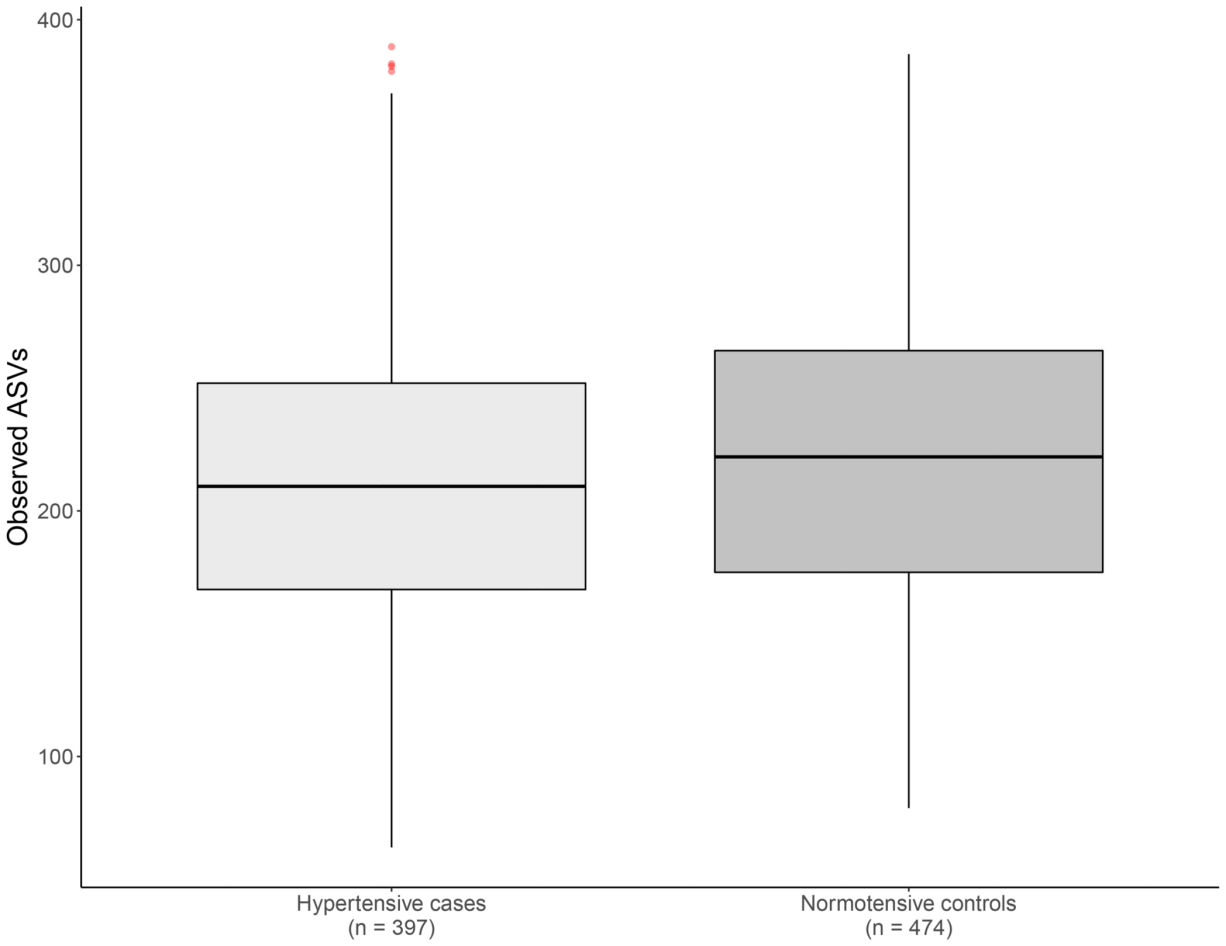
Comparison of observed ASVs within hypertensive cases and normotensive controls.

**Figure 2:**
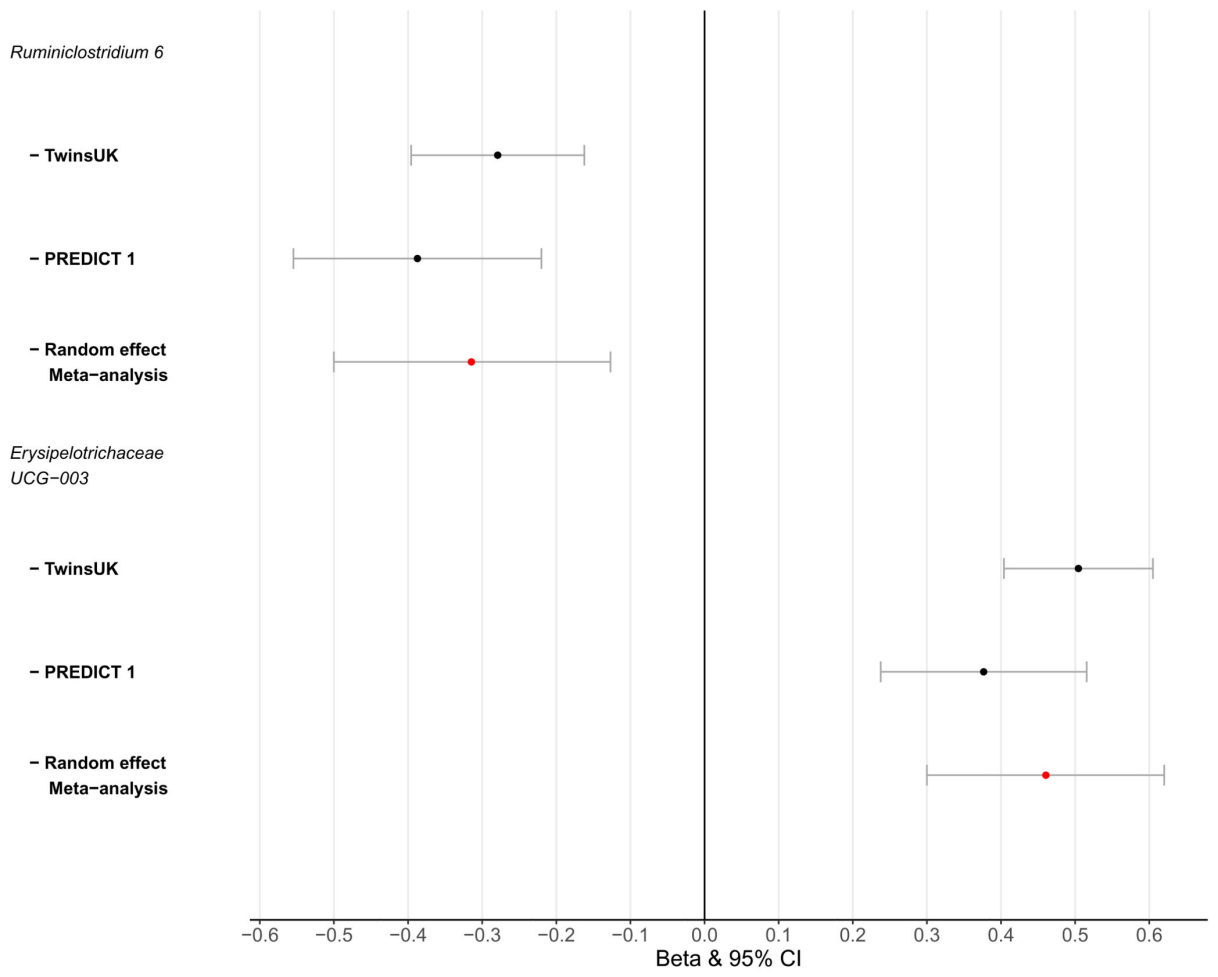
Inverse variance random effect meta-analysis of *Erysipelotrichaceae_UCG-003* and hypertension from TwinsUK and PREDICT 1.

In TwinsUK, after adjusting for confounders, we identify significantly lower observed ASVs in hypertensive subjects (Beta[95%CI] = -0.05[-0.095, -0.004], p=0.03) (Figure 1). When considering community microbial composition as measures of beta-diversity, significant differences were also detected for hypertension with binomial, Jaccard, weighted and unweighted-UniFrac diversity after multiple-testing (FDR<0.05) (Figure S1). GAMLSS models adjusted for covariates and multiple testing (FDR< 0.05) identified abundances of 10 genera significantly different in hypertensive cases compared to controls (Figure S2). These included *Coprococcus 3, Blautia, Dorea, Bifidobacterium, Erysipelotrichaceae UCG-003, Fusicatenibacter, Coprococcus 1, Ruminiclostridium 6, Subdoligranulum* and *Anaerostipes* (Figure S2). As fibre intake is recognised to influence gut microbial composition, we re-run our analysis adjusting for non-starch polysaccharide intake and results were consistent. We then assessed whether these associations were robust by testing for association of these 10 microbes in 448 independent females from the PREDICT-1 study. Out of those, 2 genera *Ruminiclostridium 6* and *Erysipelotrichacea UCG003* were nominally associated with hypertension (P<0.05) in the replication cohort (Figure 2). We then combined the results using inverse-variance random-effect meta-analysis (Figure 2 (*Ruminiclostridium 6* (meta-analysis[95%CI] = -0.31[-0.5;-0.13], p = 1x10^-3^) *Erysipelotrichacea UCG003* (meta-analysis[95%CI] = 0.46[0.3, 0.62], p=1x10^-4^).

**Figure 3:**
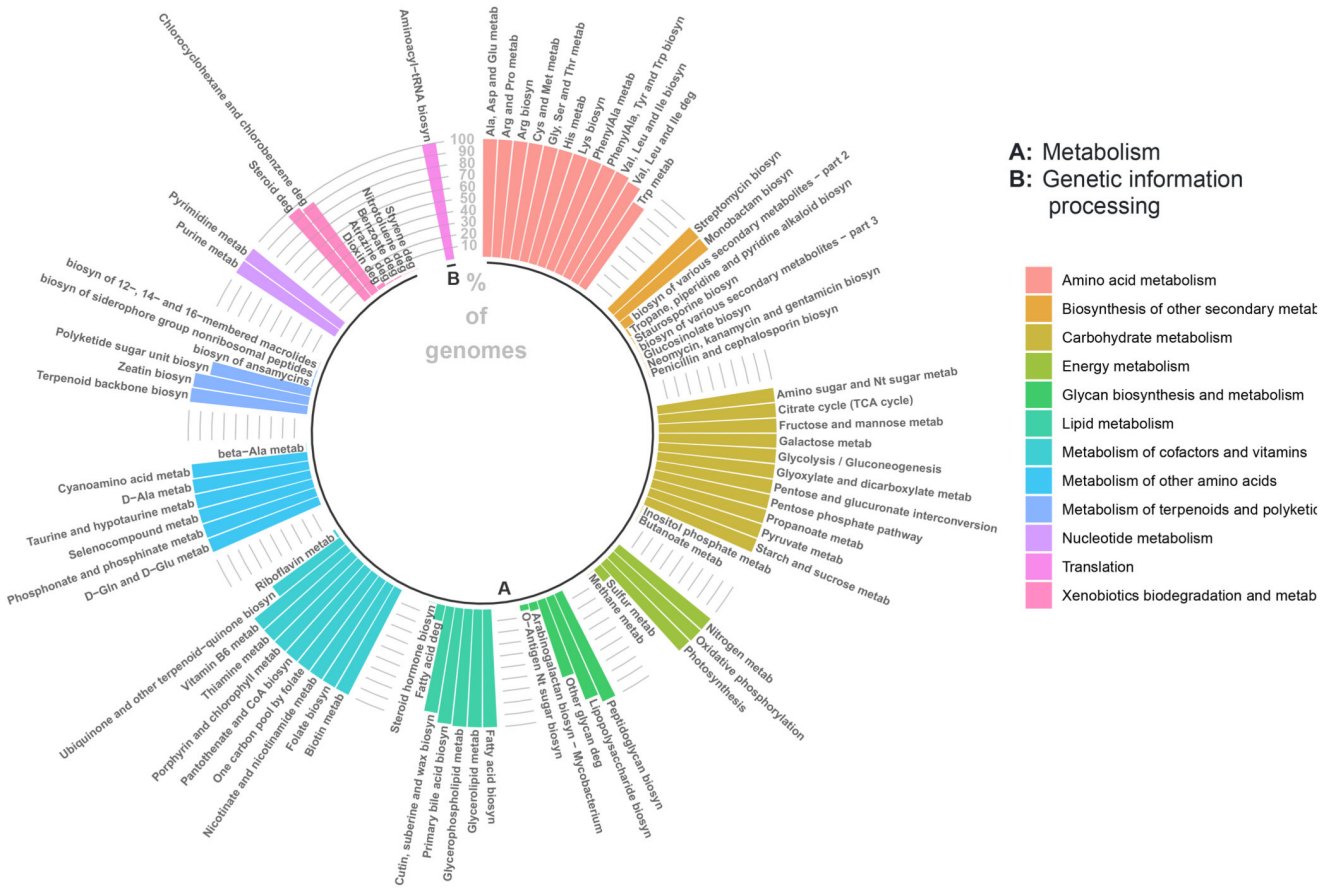
Functional genomic annotation of *Ruminiclostridium* genomes. Each bar represents the % of *Ruminiclostridium* genomic sequences that presented each KEGG pathway. The clusters are presented in the figure legend and groups symbolise processes: A, Metabolism; B, Genetic information processing.

We then investigated the functional capacity of *Ruminiclostridium* by calculating the percentage of 16s *Ruminiclostridium* genomes that presented metabolic or genetic information processing KEGG pathways. This prediction of the functional capabilities of *Ruminiclostridium* revealed that this genus might be involved in 84 KEGG pathways, 83 of which relate to metabolism (Figure 3), including lipid, amino acid, carbohydrate, cofactors and vitamin metabolism, among others (Figure 3).

**Figure 4:**
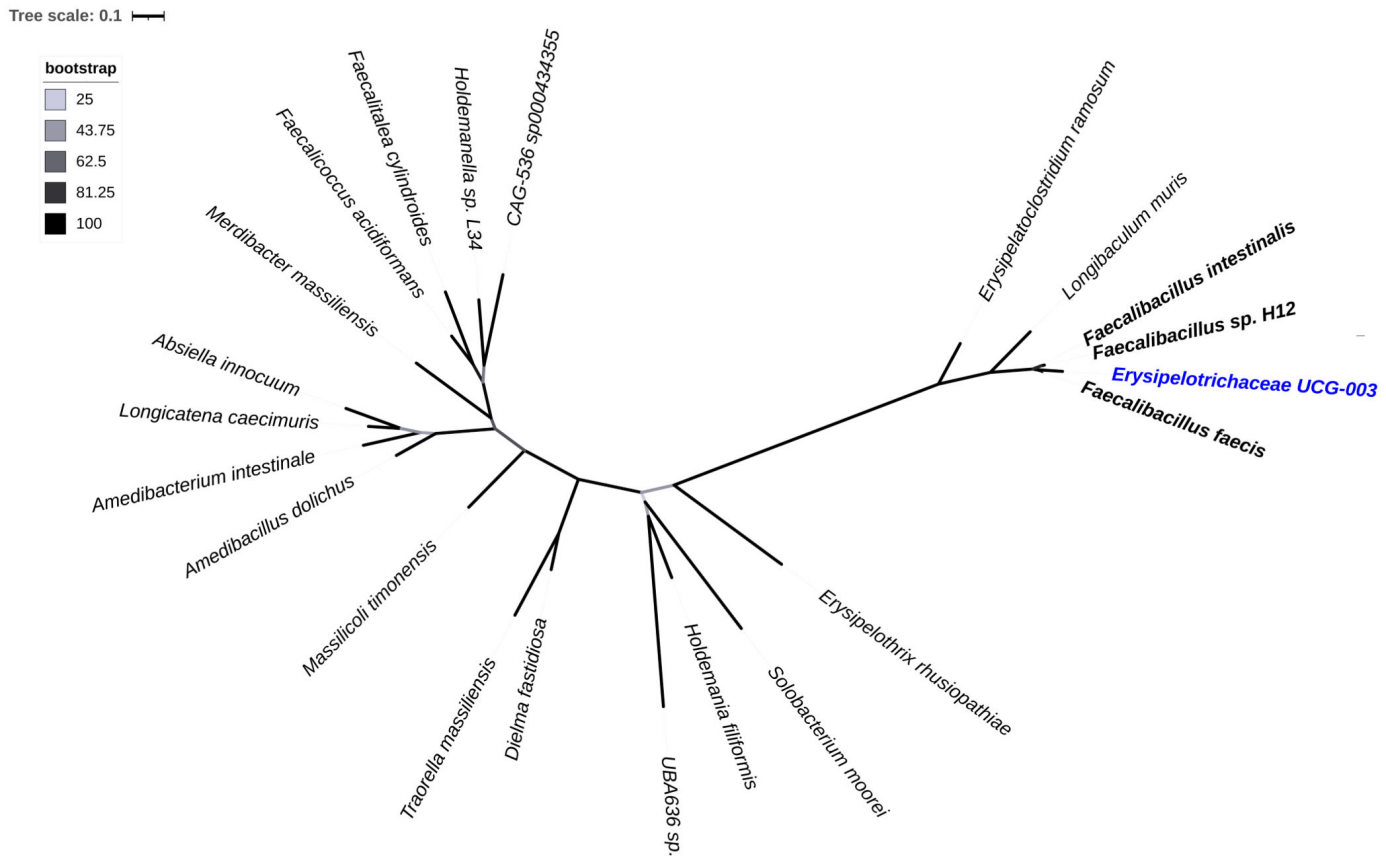
Unrooted maximum-likelihood phylogenetic tree of the 16S rRNA sequences showing the relationships of *Erysipelotrichaceae UCG-003* and the genera within the *Erysipelotrichaceae* family. Each genus is represented by a representative species, except for the *Faecalibacillus* genus, in which all the existent species are included. Only the genera isolated from human faeces, stool or the gastrointestinal tract are shown. The colour of the branches and the scale bar indicate the bootstrapping values and the average number of substitutions per site, respectively.

We then performed a genomic analysis to uncover the *Erysipelotrichaceae* genus’ evolution as it was uncultured. After constructing a maximum-likelihood phylogenetic tree using the 16S rRNA sequences of *Erysipelotrichaceae UCG-003* and the different genera within the *Erysipelotrichaceae* family, we identify that *Erysipelotrichaceae UCG-003* is closely related to existent species of the *Faecalibacillus* genus, namely, *F. intestinalis*, *F. sp. H12* and *F. faecis*, sharing the most recent common ancestor (MRCA) (Figure 4). To further interpret these results, we conducted a search using the 16S rRNA sequence of *Erysipelotrichaceae UCG-003* as a query in the RDP classifier and in BLASTn search. The RDP classifier obtained a unique match to the *Faecalibacillus* genus with 100% identity. BLASTn search reported an E-value of 2 x 10^-117^, and a 100% identity and query and query cover with sequences from the species *Faecalibacillus intestinalis* and *Faecalibacillus faecis*.

## Discussion

In this large-scale human study investigating the association between gut microbiome composition and hypertension with an independent replication, we report that gut microbiome diversity is inversely associated with hypertension in women (Figure 1, Figure S1). We also identify two genera, an uncultured genus of the *Erysipelotrichaceae* family, whose relative abundance is higher in hypertensive cases and that of *Ruminiclostridium 6*, whereby abundance was lower in hypertensive individuals (Figure 2).

Functional annotation of *Ruminiclostridium* suggests its involvement in 84 pathways (Figure 3), almost all of which related to pathways in metabolism. Moreover, the majority of the metabolic pathways were present in all genomic sequences (Figure 3), implying a high homogeneity in the metabolic functional capabilities among the different species of *Ruminiclostridium*. A number of these pathways have been previously implicated in BP regulation. For instance, tryptophan biosynthesis and metabolism has been linked to SBP, [3]; while thiamine metabolism, has also been correlated to hypotension in murine models [42] and thiamine supplement studies [43] (Figure 3).

Through evolutionary analysis of the uncultured *Erysipelotrichaceae* genus, we identify a close relationship, and shared MRCA with *F. sp. H12*, *F. faecis* and *F. intestinalis*, species of *Faecalibacillus* (Figure 4). Implying that *Erysipelotrichaceae UCG-003* belongs to or has evolved from *Faecalibacillus*. Subsequent results obtained from the RDP classifier and BLASTn search support that *Erysipelotrichaceae UCG-003* does in fact belong to *Faecalibacillus* with a 100% identity match and E-value of 2 x 10^-117^.

*Faecalibacillus* is a Gram-positive genus [44], first isolated from healthy South Korean subjects[45]. Due to the novelty of this genus and lack of phenotypic characterisation it was not included in the latest SILVA database, from which we assigned taxonomic ranks [45,46].

Animal models and case studies of patients have shown that members of the *Erysipelotrichaceae* family are significantly increased in a number of inflammatory conditions [47], such as irritable bowel syndrome [48], colorectal cancer [47], and rheumatoid arthritis with anti-citrullinated protein autoantibodies [49], suggesting a pro-inflammatory effect of this bacterial family on the host. Previous studies report an association between abundance of *Erysipelotrichaceae* family members and host lipidemic profile, particularly relating to cholesterol levels [47]. However, after adjusting our analysis for levels of total cholesterol, the association between hypertension and *Faecalibacillus* remained statistically significant.

Our study has some limitations. Firstly, the study was based on middle aged white female twins and hence may not be generalisable to other ethnic groups or to men. Although, the characteristics of these women are representative of the general UK female population [23], clearly studies in men and in other ethnic groups are needed. Secondly. the cross-sectional nature of our data does not allow us to infer causality. Third, although our genomic analysis highlights the functional capabilities of *Ruminiclostridium*, it does not provide information on which pathways are actually active. Therefore, more research is required to comprehensively understand the mechanisms by which *Ruminiclostridium* can influence hypertension. However, our study does benefits from our large sample size, facilitating the analysis of numerous microbes with sufficient power and an independent replication.

In conclusion, we find that gut microbiome diversity and composition are associated with hypertension. Our results suggest that targeting the microbiome may be a novel way to prevent or treat hypertension. Foremost, more research is necessary to further corroborate correlations between the gut microbiome and hypertension and provide insights into mechanistic capacity of the gut microbiome to control BP.

## Supplementary Material

Supplementary figure 1 MDS/PCoA of weighted-Unifrac distance & hypertension in the TwinsUK cohort with normal and multivariate t-distributed ellipses.

Supplementary figure 2 Bacterial genera correlated with hypertension in the TwinsUK cohort.

## Figures and Tables

**Table 1:** Descriptive characteristics of TwinsUK and PREDICT1 samples.

	TwinsUK (n = 871)				PREDICT (n = 448)			
	Cases (n = 397)		Controls (n = 474)		Cases (n = 57)		Controls (n = 391)	
	*n*	*%*	*n*	*%*	*N*	*%*	*n*	*%*
**Female**	397	100	474	100	57	100	391	100
	*Mean*	*SD*	*Mean*	*SD*	*Mean*	*SD*	*Mean*	*SD*
**Age** (yrs)	60.33	8.72	52.41	11.9	50.27	11.72	43.97	11.99
**BMI** (Kg/m^2^)	28.14	5.41	24.19	3.77	27.9	6.64	24.63	4.66
**SBP** (mmHg)	138.98	15.01	109.24	6.76	122.95	15.39	102.12	8.31
**DBP** (mmHg)	83.48	10.14	69.05	6.21	84.43	9.83	69.94	6.07

## Abbreviations

BP: blood pressure
SBP: systolic blood pressure
DBP: diastolic blood pressure
ASV: amplicon sequence variants
GAMLSS: generalized additive model for location, scale and shape
BEZI: zero-inflated beta distribution
KEGG: Kyoto Encyclopedia of Genes and Genomes
MRCA: most recent common ancestor
