# Supplementary material for: Gut microbiome diversity and composition is associated with hypertension in women
Source: J Hypertens. 2021 May 10;39(9):1810–6. doi: 10.1097/HJH.0000000000002878 (PMC7611529; doi:10.1097/HJH.0000000000002878)
Supplement: Supplemental Digital Content [file jhype-39-1810-s001.docx]

**Figure S1.** MDS/PCoA of weighted-Unifrac distance & hypertension in the TwinsUK cohort with normal and multivariate t-distributed ellipses.
