# Supplementary material for: Gut microbiome diversity and composition is associated with Hypertension in women
Source: J Hypertens. Author manuscript; Available in PMC 2021 Sep 1. (PMC7611529; doi:10.1097/HJH.0000000000002878)
Supplement: Supplementary file 1 [file EMS121504-supplement-Supplementary_figure_1.pdf]

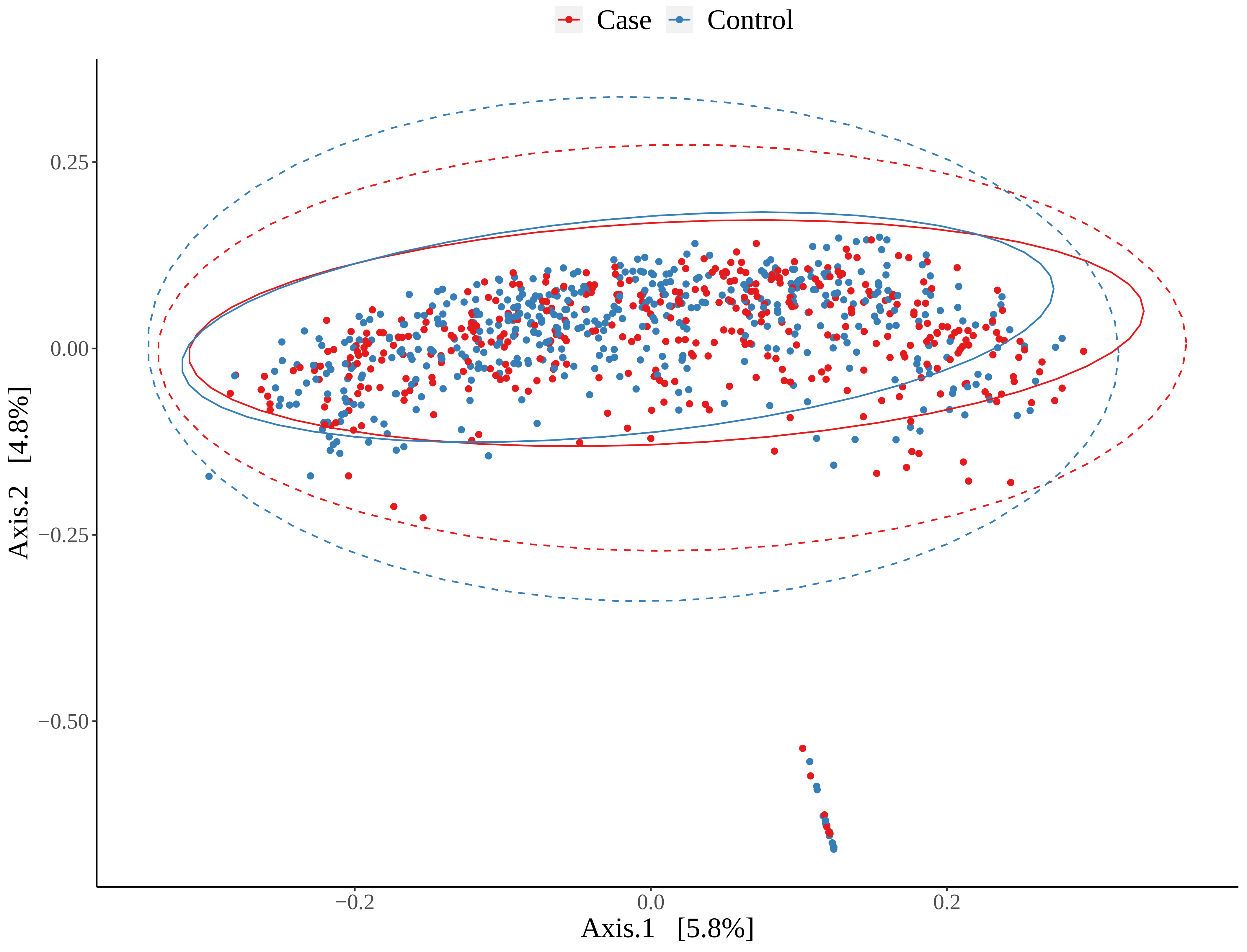

Case
Control
0.25
0.00
−0.25
−0.50
Axis.2 [4.8%]
−0.2
0.0
0.2
Axis.1 [5.8%]
