# Supplementary material for: Gut microbiome diversity and composition is associated with Hypertension in women
Source: J Hypertens. Author manuscript; Available in PMC 2021 Sep 1. (PMC7611529; doi:10.1097/HJH.0000000000002878)
Supplement: Supplementary file 2 [file EMS121504-supplement-Supplementary_figure_2.pdf]

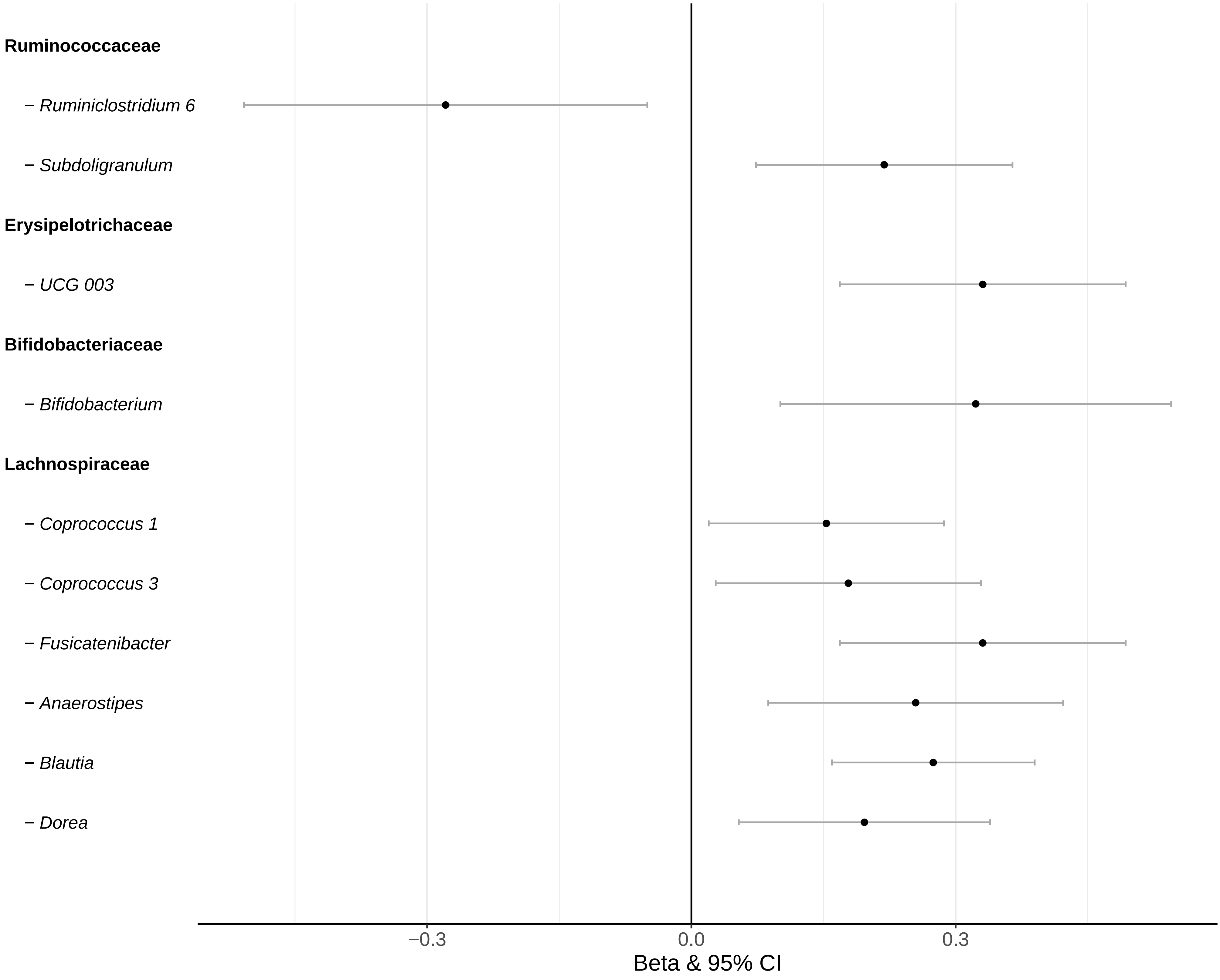

Ruminococcaceae
− Ruminiclostridium 6
− Subdoligranulum
Erysipelotrichaceae
− UCG 003
Bifidobacteriaceae
− Bifidobacterium
Lachnospiraceae
− Coprococcus 1
− Coprococcus 3
− Fusicatenibacter
− Anaerostipes
− Blautia
− Dorea
−0.3
0.0
0.3
Beta & 95% CI
